# Effect of a Habitual Late-Evening Physical Task on Sleep Quality in Neither-Type Soccer Players

**DOI:** 10.3389/fphys.2018.01582

**Published:** 2018-11-06

**Authors:** Jacopo A. Vitale, Giuseppe Banfi, Antonio La Torre, Matteo Bonato

**Affiliations:** ^1^IRCCS Istituto Ortopedico Galeazzi, Milan, Italy; ^2^Vita-Salute San Raffaele University, Milan, Italy; ^3^Department of Biomedical Sciences for Health, Università degli Studi di Milano, Milan, Italy

**Keywords:** sleep, soccer, chronotype, habitual training time, actigraphy, orthopedics

## Abstract

**Purpose:** The aim of this study was to investigate objective and subjective sleep quality, daytime tiredness and sleepiness in response to a late-evening high intensity interval training (HIIT) session in neither-type soccer players that habitually trained late in the day. This is the first study that considered both athletes’ chronotype and habitual training time as crucial factors when assessing sleep quality in relation to an evening physical task.

**Methods:** In this longitudinal, prospective, observational study, 14 Italian soccer players were recruited (mean age: 26.1 ± 4.5 years; height: 1.81 ± 0.06 m; weight: 78.9 ± 6.1 kg) and performed an extra-routine 4 × 4-min HIIT session at 09:00 p.m. Players used to train always between 09:00 and 11:00 p.m during the competitive season. All subjects wore an actigraph to evaluate their objective sleep parameters and a sleep diary was used to record subjective values of sleep quality, daytime tiredness, and daytime sleepiness. All data were analyzed as: the mean of the two nights before (PRE), the night after (POST 1), and the mean of the two nights after (POST 2) the extra-routine HIIT session. The subjects’ chronotype was assessed by the morningness-eveningness questionnaire (MEQ).

**Results:** All players were classified as N-types (mean MEQ score: 49.4 ± 3.7). None of the actigraph parameters nor the subjective values of sleep quality, tiredness, and sleepiness showed significant changes in PRE, POST 1, and POST 2.

**Conclusion:** The results of our study added more information regarding sleep quality outcomes in response to a late-evening HIIT session. Athletic trainers and medical staff should always control for chronotype and habitual training time when assessing variations to sleep quality in athletes.

## Introduction

Success for athletes is determined by several factors, such as physical and mental training, nutrition, muscle and bone health, and also by the ability to properly recover and rest between training sessions and competitions. In this context, sleep plays an equally important role (Knufinke et al., 2018). Actually, athletes’ sleep is often disturbed and it is seems challenging to reach optimal levels of sleep quality and quantity (Leeder et al., 2012). Indeed, long travels, different sleeping environments, anxiety, elevated training loads, and many other factors are able to negatively influence the nocturnal rest (Roy and Forest, 2018). Late-night competitions or training sessions could be tricky too for athlete’s sleep but results are controversial: it was reported that late-night soccer training did not affect athletes’ nocturnal heart-rate-variability (Costa et al., 2018) or sleep behavior (Robey et al., 2014) while, on the contrary, Vitale et al. (2018) showed that elite volleyball players worsened their sleep quality and perceived recovery following a night game. This discrepancy could be due to confounders that have not been well controlled and that possibly could have influenced players’ sleep quality. Chronotype could play a key role for sleep: it has been reported that morning-type (M-type) soccer players worsened their sleep quality in response to an evening high intensity interval training (HIIT) session while no differences were detected for evening-type (E-type) players (Vitale et al., 2017). Furthermore, the habitual training time need to be considered when assessing the physiological responses to an evening performance: athletes who habitually trained in the evening, beyond their chronotype, reported lower ratings of perceived exertion in the evening, with better performance results, compared to a morning session (Rae et al., 2015), however, this variable has never been studied in relation to the athlete’s sleep. Therefore, the aim of this study was to assess objective and subjective sleep quality, subjective daytime tiredness, and subjective daytime sleepiness in response to a late-evening HIIT session in neither-type (N-type) soccer players that habitually trained at 09:00–11:00 p.m. To the best of our knowledge, this is the first study that considered both the athletes’ chronotype and the habitual training time-of-day as crucial factors when studying sleep quality in relation to an evening physical performance. Our rationale for selecting only neither-type soccer players was to avoid confounding results due to the chronotype effect (i.e., morningness and eveningness effects on sleep behavior). We hypothesized to not observe significant decrement in sleep, tiredness, and sleepiness parameters in N-type players.

## Methods

### Study Design and Subjects

This was a longitudinal, prospective, observational study. Soccer players were recruited from a non-professional Italian male team of the seventh league of Federazione Italiana Giuoco Calcio. Players’ chronotype was assessed by the morningness-eveningness questionnaire (MEQ; Horne and Ostberg, 1976). According to the MEQ-score, participants were categorized as M-types (scoring ≥ 59), E-types (scoring ≤41), or neither-type (N-types, scoring 42–58). Inclusion criteria were age ≥ 18 years, and soccer practice of three times a week for ≥2 h in the evening. Exclusion criteria were being a goalkeeper, being a morning- or evening-type, tobacco use, and medical conditions contraindicating physical exercise. Before entering the study, all participants provided written informed consent to the experimental procedure, which was previously approved by the Ethical Committee of Università degli Studi di Milano in compliance with current laws and regulations governing the use of human subjects (Declaration of Helsinki II).

### Late-Evening High Intensity Interval Training Protocol

Soccer players, during the competitive season, used to train at the same time of the day, between 09:00 and 11:00 p.m., three times per week, on Tuesday, Wednesday, and Friday. All players, in April 2018, performed an extra-routine HIIT session at 09:00 p.m., on Thursday. During the test, heart rate was recorded using a heart rate monitor (Polar RS800, Polar, Kempele, Finland). The HIIT protocol was the 4 × 4-min interval training (Helgerud et al., 2001) and it consisted of running 4 times at 90–95% of HR_max_ with 3 min of active recovery at 70% of HR_max_ between each interval.

### Actigraphy and Subjective Evaluations

All players wore an actigraph (Actiwatch 2, Philips Respironics, OR, United States) to record their sleep parameters. The actigraph monitoring lasted from Monday to Friday. A high actigraphic sensitivity threshold was used (80 counts) since it provides the best combination of sensitivity and specificity when studying sleep in athletes (Sargent et al., 2016). A sleep diary was used to record bed time, wake up time, hours napping, and subjective values of sleep quality, daytime tiredness, and daytime sleepiness. Data derived from actigraphs and sleep diaries were used to determine the amount and quality of sleep participants obtained. The description of both actigraphy-based and subjective parameters is reported in Table 1.

**Table 1:** Description of the six sleep parameters evaluated through actigraphy and the three subjective parameters of sleep quality, daytime tiredness, and daytime sleepiness in soccer players.

Objective and subjective sleep parameters	Parameters description
Time in Bed *(TB, minutes)*	The amount of time spent in bed attempting to sleep between bedtime and get up time.
Sleep Latency *(SL, minutes)*	The period of time between bedtime and sleep onset time.
Sleep Efficiency *(SE, %)*	The percentage of time in bed that was spent asleep.
Wake After Sleep Onset *(WASO, min)*	The amount of time spent awake after sleep has been initiated.
Immobility Time *(IT, %)*	The total time, expressed in percentage, spent without recording any movement during time in bed.
Fragmentation Index *(FI, %)*	Sum of mobility and immobility accesses in 1 min, divided by the number of immobility accesses.
Subjective Sleep Quality *(1-to-10 point scale)*	Subjective value of sleep quality in a scale from 0 = very poor sleep quality, to 10 = optimal sleep quality.
Subjective Daytime Tiredness *(1-to-10 point scale)*	Subjective value of daytime tiredness in a scale from 0 = not at all tired, to 10 = extremely tired.
Subjective Daytime Sleepiness *(1-to-10 point scale)*	Subjective value of daytime sleepiness in a scale from 0 = not at all sleepy, to 10 = extremely sleepy.


Objective and subjective sleep quality data and subjective parameters of tiredness and sleepiness were analyzed as: the mean of the two nights before (PRE), the night after (POST 1), and the mean of the two nights after (POST 2) the extra-routine HIIT session.

### Statistical Analysis

Data are presented as mean ± standard deviation (SD). Each objective and subjective sleep parameter were checked with the Shapiro–Wilk test at PRE, POST 1, and POST 2. Repeated-measures analysis of variance (RM-ANOVA) followed by the Bonferroni *post-hoc* test, or Friedman test with Dunn’s comparison for non-normal distributed variables, was performed to test the differences in sleep among PRE, POST 1, and POST 2. A *p*-value ≤0.05 was considered statistically significant.

## Results

**FIGURE 1:**
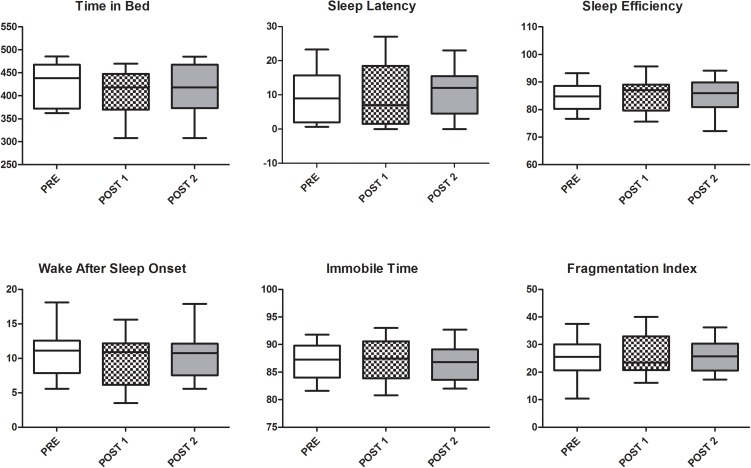
Whiskers plot with median, first and third quartiles, and minimum and maximum values of the six actigraphy-based sleep parameters in PRE, POST 1, and POST 2.

**FIGURE 2:**
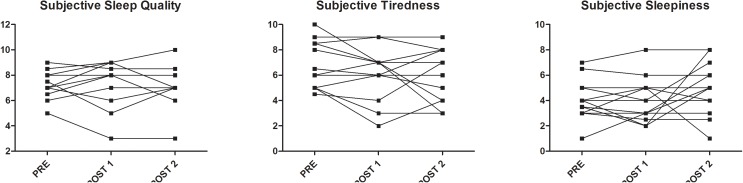
Raw data, with connecting lines, of subjective sleep quality, tiredness, and sleepiness in PRE, POST 1, and POST 2.

Twenty-one soccer players were screened and seven were excluded because E-types (*N* = 2), injured (*N* = 3), or goalkeepers (*N* = 2). Therefore, 14 soccer players were finally recruited (age: 26.1 ± 4.5 years; height: 1.81 ± 0.06 m; body mass: 78.9 ± 6.1 kg, MEQ score: 49.4 ± 3.7). The intensity of HIIT session was 175 ± 5 bpm, corresponding to 92 ± 3% of HR_max_. All soccer players were classified as N-types. None of the actigraph parameters nor the subjective values of sleep quality, tiredness, and sleepiness showed significant changes in PRE, POST 1, and POST 2. Figure 1 shows the Whiskers plot of the six actigraphy-based parameters while Figure 2 reports the raw data, with connecting lines, of subjective values of sleep quality, tiredness, and sleepiness.

## Discussion

The study highlighted no differences in objective and subjective sleep quality in response to an extra-routine late-evening HIIT session in N-type soccer players accustomed to train late in the day. In addition, neither subjective values of daytime tiredness nor subjective daytime sleepiness showed significant variations. Our initial hypothesis was confirmed.

The strength of this study is that we controlled for two important confounding factors, both able to influence athlete’s sleep in response to a physical task: the chronotype and the habitual training time. The athletes’ chronotype was determined by the MEQ and all included players were N-types; M- and E-types were excluded from this trial since these two chronotype categories showed large differences in their sleep behavior: M-types usually registered higher sleep quality and quantity in normal conditions but they suffered more the influence of an evening training session while, conversely, E-types can more easily adapt to delayed training times without showing worsening of their sleep quality (Vitale and Weydahl, 2017; Vitale et al., 2017). Having a homogenous study sample, with subjects belonging to the same intermediate category of chronotype, have favored a clearer interpretation of sleep data. In addition, the “habitual training time” variable was controlled too since all soccer players were accustomed to train late in the evening (09:00–11:00 p.m.). It has been recently showed that diurnal variations in the psychophysiological responses to a performance are also related to the players’ habitual training time: athletes who habitually trained in the evening had lower ratings of perceived exertion and better performances in the evening compared to a morning session and, similarly, athletes who habitually trained in the morning had lower fatigue and higher vigor scores prior to a morning physical performance (Rae et al., 2015). It is therefore possible that, in the present study, the habitual (evening) training time was able to mitigate the potential negative effects on sleep dictated by a high-intensity evening physical task. However, further studies to be conducted with a cross-over design, including both M-, N-, and E-type athletes, are needed to confirm these preliminary data.

The present findings could help coaches to better interpret the discrepancies that have been previously observed in sleep behavior with reference to evening physical tasks; the chronotype and the habitual training time should be always considered by athletic trainers and medical staff when assessing variations to sleep quality in athletes. Avoiding sleep problems represents a key strategy of primary prevention for muscle and bone injuries.

## Author Contributions

JV and MB participated in the data collection and analysis. JV conducted the manuscript writing process. All authors were involved during study set-up and done final revision.

## Conflict of Interest Statement

The authors declare that the research was conducted in the absence of any commercial or financial relationships that could be construed as a potential conflict of interest.
